# Mexican-Hat-Like Response in a Flexible Tactile Sensor Using a Magnetorheological Elastomer

**DOI:** 10.3390/s18020587

**Published:** 2018-02-14

**Authors:** Takumi Kawasetsu, Takato Horii, Hisashi Ishihara, Minoru Asada

**Affiliations:** 1 Department of Adaptive Machine Systems, Graduate School of Engineering, Osaka University, Suita 5650871, Japan; ishihara@ams.eng.osaka-u.ac.jp (H.I.); asada@ams.eng.osaka-u.ac.jp (M.A.); 2 Department of Mechanical Engineering and Intelligent Systems, Graduate School of Informatics and Engineering, The University of Electro-Communications, Chofu 1828585, Japan; takato@uec.ac.jp

**Keywords:** force and tactile sensing, tactile sensor, robotic skin, flexible materials, magnetorheological elastomer, magnetic flux measurements

## Abstract

A significant challenge in robotics is providing a sense of touch to robots. Even though several types of flexible tactile sensors have been proposed, they still have various technical issues such as a large amount of deformation that fractures the sensing elements, a poor maintainability and a deterioration in the sensitivity caused by the presence of a thick and soft covering. As one solution for these issues, we proposed a flexible tactile sensor composed of a magnet, magnetic transducer and dual-layer elastomer, which consists of a magnetorheological and nonmagnetic elastomer sheet. In this study, we first investigated the sensitivity of the sensor, which was found to be high (approximately 161 mV/N with a signal-to-noise ratio of 42.2 dB); however, the sensor has a speed-dependent hysteresis in its sensor response curve. Then, we investigated the spatial response and observed the following results: (1) the sensor response was a distorted Mexican-hat-like bipolar shape, namely a negative response area was observed around the positive response area; (2) the negative response area disappeared when we used a compressible sponge sheet instead of the incompressible nonmagnetic elastomer. We concluded that the characteristic negative response in the Mexican-hat-like response is derived from the incompressibility of the nonmagnetic elastomer.

## 1. Introduction

The sense of touch provides essential information for robots working in an unknown environment, handling unknown objects and interacting with humans. A number of researchers has been proposing various types of flexible tactile sensors with safe, flexible and protective coverings intended to provide tactile sensations for robots, e.g., [1,2,3]. However, various technical issues remain, such as a large amount of deformation that causes the sensing elements to fracture, deterioration in the sensitivity caused by the thick and soft coverings and a poor maintainability.

**Figure 1:**
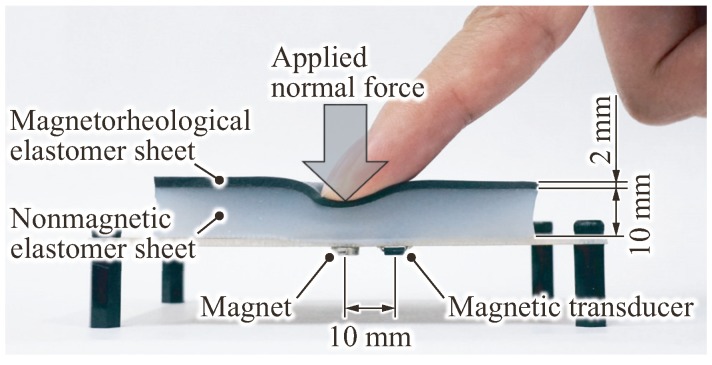
Basic structure of the proposed flexible tactile sensor, which can detect an applied normal force and vertical deformation (modified from [4]).

To address the above issues in conventional flexible sensors, we proposed and developed a magnetic flexible tactile sensor [4,5,6], shown in a minimal configuration in Figure 1. The proposed sensor has four advantages: (1) the flexible covering contains no solid parts, sensing elements or wiring; (2) the sensor has high sensitivity and extremely low rigidity with respect to the surface deformation of its flexible covering; (3) the electronic and fragile elements in the sensor are completely separated from the parts to which the contact forces are applied; and (4) the flexible covering can be replaced when it becomes damaged and requires no precise positioning.

In the previous study [5], we investigated the sensor response versus contact force, and we found that the sensor can measure the applied normal force with a specific low contact speed (0.1 mm/s). However, the sensor response curve could be changed by the contact speed because a higher contact speed causes a change in the deformation behavior of the elastomer. Therefore, the sensor response should be investigated with higher contact speeds, which may be present in a real task. In addition, the spatial response of the sensor should be investigated to determine an appropriate spatial layout for the proposed sensors for large-area implementation. This is because the complex deformation of the elastomer and the complex behavior of the change in the magnetic field make it difficult to predict the spatial response of the sensor.

In order to investigate these sensor characteristics, we measured the sensor responses with different contact speeds and the spatial responses in this study. The results can be summarized as follows: (1) the sensitivity of the sensor was found to be high (approximately 161 mV/N with a signal-to-noise ratio of 42.2 dB) among flexible tactile sensors with a thick and soft covering; (2) a higher contact speed causes the response to remain after applying a load, and this remaining response was larger at a higher speed; (3) the sensor has a distorted Mexican-hat-like bipolar spatial response, and this bipolar spatial response can be practically fit with an elliptical difference-of-Gaussians function whose major axis corresponds to the line connecting the centers of the magnet and magnetic transducer; and (4) the sensor basically has an elliptical Gaussian-like unipolar spatial response versus the applied load; however, the incompressibility of the employed dual-layer elastomer adds a negative response to this unipolar response, and the entire spatial response exhibits a Mexican-hat-like bipolar spatial response.

The remainder of this article is organized as follows. Section 2 provides a review of conventional tactile sensors and summarizes the open issues. The proposed tactile sensor is then introduced in Section 3. Section 4 provides the methods and results of the experiments. These results and the fitting function of the proposed sensor are then discussed in Section 5. Finally, the conclusions and future issues are presented in Section 6.

## 2. Related Works

**Figure 2:**
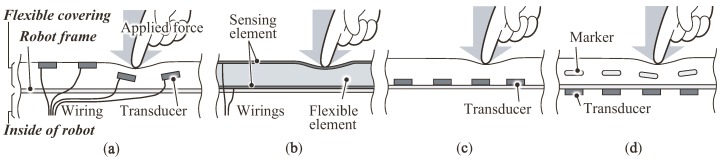
Schematics of the structures of conventional flexible tactile sensors holding a thick flexible contact layer: (**a**) type A; (**b**) type B; (**c**) type C; (**d**) type D.

Since various types of tactile sensors have been proposed (see the reviews [3,7,8]), this section summarizes the issues with conventional flexible tactile sensors covered with a thick flexible and soft contact layer. We first define three kinds of parts consisting of a flexible tactile sensor: a flexible covering, the robot frame and the inside of the robot (illustrated in Figure 2a). The flexible covering is the outermost part where a contact force is applied. The robot frame holds the flexible covering and protects the inside of the robot. Tactile transducers are placed either into or onto these three parts as follows.

- Type A, Transducers are placed into a flexible covering: This type of sensor (Figure 2a; e.g., [9,10,11,12]) places the transducers with the associated electrical wiring inside or on the surface of the flexible covering. In this structure, the transducers directly capture the deformation or vibration of the covering. One of the issues of this type of structure is that the transducers inside or on the surface of the covering deteriorate the durability of the sensor because a large stress and shock can be added onto the delicate transducers. In addition, Goka et al. [13] have pointed out that the wiring in the flexible covering might be damaged with a large amount of deformation of the covering; therefore, they developed a magnetic tactile sensor (we classify this sensor here as Type D, as described below). Recent progress in stretchable electronics techniques [14,15,16] for wiring may solve this issue.
- Type B, The flexible covering functions as a transducer: In this approach, sensors employ a flexible covering that functions as a transducer, e.g., using capacitive or resistive technology [2,17,18,19]. The transducer consists of a flexible functional element inserted between a pair of sensing elements such as a stretchable electrode. These sensors can measure the applied load with a high sensitivity since the transducers capture the surface deformation directly. A large number of wires, which connect the flexible covering and the inside of the robot, might diminish the maintainability.
- Type C, Transducers are mounted onto the surface of the robot frame: These sensors (Figure 2c; e.g., [1,20,21,22,23]) mount their transducers onto the outer surface of the robot frame. Because their flexible covering has no wiring or transducers, there are no problems with wiring disconnection, and replacement of the covering is easy. Shimojo [24] has pointed out that a flexible covering functions as a spatial low-pass filter for a tactile transducer when the sensor elements are under the covering. Therefore, the sensitivity will be diminished by a thick and soft covering such as a compressible sponge sheet.
- Type D, Transducers are placed inside the robot with their markers inside the covering: For this type of sensor (Figure 2d), the transducers can be removed from the part where a contact force is applied; instead, the transducers are placed inside the robot. The applied loads are estimated by monitoring the displacement of the markers embedded inside the covering.

An example of this type is a magnetic tactile sensor (e.g., [13,25,26,27,28]) employing magnetic technology. In these sensors, magnetic transducers are placed inside the robot, and magnets are placed into the flexible covering as their magnetic markers. To measure the applied forces, these sensors detect the changes in the magnetic field caused by the displacement of the magnet. One issue with this approach is that replacement of the covering containing the magnets requires precise positioning with the corresponding magnetic transducers because their positional relationship determines the response.

A similar configuration can be implemented by using an image sensor with color markers [29,30,31]. This type of sensor can also separate the transducers from the flexible covering. Because these sensors require an optical system, their structure will be complex and difficult to miniaturize.

In summary, flexible tactile sensors have several issues such as fracture of the sensing elements by a large amount of deformation, deterioration in the sensitivity caused by the thick and soft covering and poor maintainability. Our proposed flexible tactile sensor can provide one solution for these issues.

## 3. Proposed Tactile Sensor

We developed a magnetorheological elastomer tactile sensor [6] that overcomes the issues described in the previous sections. This section provides a brief explanation of how these issues can be solved.

### 3.1. Structure and Sensing Mechanism

**Figure 3:**
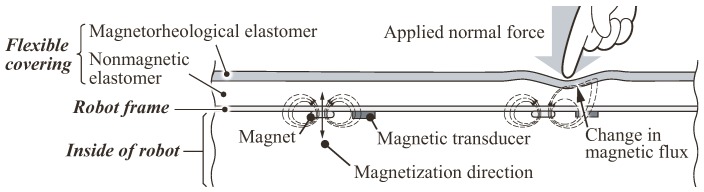
Schematic of the structure and sensing mechanism of the proposed sensor.

Figure 3 illustrates the structure and sensing mechanism of the proposed sensor. The sensor is composed of a flexible dual-layer elastomer, a robot frame holding a permanent magnet and a magnetic transducer, which responds to the amount of magnetic flux penetrating its body. As described in Figure 3, left, the magnetization of the magnet is in a direction perpendicular to the dual-layer elastomer. The lower layer of the elastomer is a nonmagnetic base elastomer (BE) sheet, whereas the upper one is a magnetorheological elastomer (MRE) sheet, which contains particles with a high magnetic permeability, such as iron particles.

In the absence of a contact force, a certain amount of magnetic flux generated by the magnet penetrates the elastomer and magnetic transducer, as shown in Figure 3, left. The force applied to the elastomer surface deforms the top MRE sheet and causes a decrease in the distance between the MRE and the transducer. This distance determines the amount of magnetic flux penetrating the transducer because the magnetic permeability around the transducer is increased by approaching the MRE (Figure 3, right). As a consequence, the applied force can be estimated by the amount of magnetic flux penetrating the transducer.

From this structure, the sensor response can be determined by the following parameters [5,6]: (1) the thickness of the BE (a thicker BE results in a larger sensing range, while the sensitivity becomes lower); (2) the thickness of the MRE (a thicker MRE results in a higher sensitivity); (3) the magnetic field strength generated by the magnet (a stronger magnetic field produces a larger sensor response); and (4) the distance between the magnet and the magnetic transducer (a shorter distance produces a larger sensor response).

### 3.2. Fabricated Sensor

**Figure 4:**
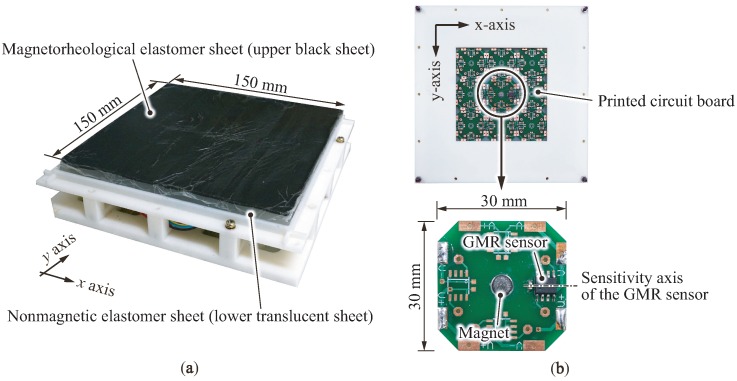
Overview of the fabricated tactile sensor used in this study: (**a**) Appearance of the sensor; (**b**) Bottom view.

The appearance and an overview of the proposed sensor are shown in Figure 4a,b. The dual-layer elastomer is 150 mm long, 150 mm wide and 12 mm thick in which a 2 mm-thick MRE sheet is laminated onto a 10 mm-thick BE sheet. Both elastomer layers are made of a low-rigidity platinum-cured elastomer (Smooth-on Inc., Macungie, PA, USA, Dragon Skin FX Pro), which is compounded with a plasticizer (Smooth-on Inc., Macungie, PA, USA, Dragon Skin Thinner) with a mass ratio of 200%. In this study, we employed carbonyl iron particles (BASF Corp., Ludwigshafen, Germany, hard grade HQ type) with a particle diameter of 2 μm at a volume ratio of 20%. To make the MRE sheet, we mixed iron particles in the platinum-cured elastomer before curing and poured it into a rectangular female mold until curing.

A disk-shaped neodymium magnet, whose magnetization direction is its axial direction, is mounted at the center of the underside of the printed circuit board (PCB). The diameter and thickness of the magnet are 5 and 1.5 mm, respectively, and its surface magnetic flux density is 0.2 T. As a magnetic transducer, we employ a giant magnetoresistance (GMR) sensor (NVE Corp., Eden Prairie, MN, USA, AA003-02E), which offers a high sensitivity to a slight change in the magnetic field. The output of the GMR sensor is determined by the amount of the magnetic flux penetrating its body with a sensitivity of 0.1 V/mT (at a supply voltage of 5 V). The GMR sensor is mounted 10 mm from the magnet on the underside of the PCB, as the direction of its sensitivity axis is toward the magnet (i.e., the dashed line on the right side of Figure 4b. In this study, we mounted only one magnet and one GMR sensor (Figure 4b, right), even though the PCB could hold many magnets and GMR sensors (planned for future work with a large sensing area). Here, we focus on the sensor response curve and spatial response of a single pair of sensors, i.e., one magnet and one GMR sensor.

## 4. Experiments

**Figure 5:**
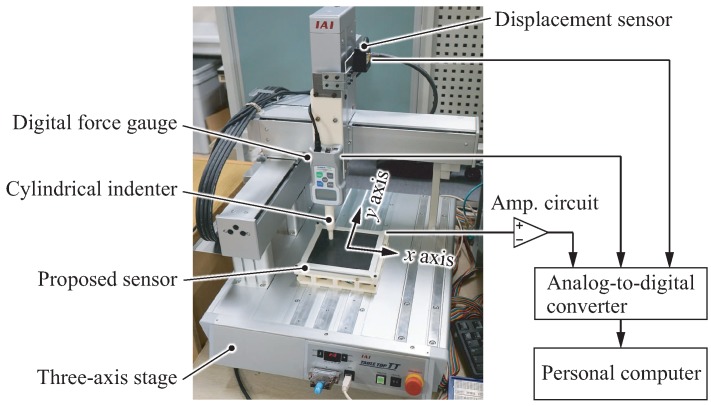
Experimental setup for investigating the sensor response.

Figure 5 shows the experimental setup for investigating the sensor response curve and spatial response. The proposed sensor was mounted to a three-axis robot stage (IAI Corp., Shizuoka, Japan, TTA-C3-WA-30-25-10) holding a digital force gauge (Nidec Corp., Kyoto, Japan, FGP-5) with a measurement force resolution of 0.01 N and a temporal resolution of 1 ms. The tip of the force gauge was equipped with a plastic cylindrical indenter with a diameter of 10 mm. To measure the vertical displacements precisely, the stage was also equipped with a laser displacement sensor (Omron Corp., Kyoto, Japan, ZX0-LD100A61) with a resolution of 0.08 mm.

The output voltage of the GMR sensor was amplified by an amplification circuit with a gain of 200. A personal computer captured the outputs of the amplified sensor, force gauge and displacement sensor via an analog-to-digital converter (CONTEC Corp., Osaka, Japan, ADI12-8GY with CPU-CA20GY) with a sampling rate of 200 Hz.

### 4.1. Sensor Response and Contact Speed

To investigate how the contact speed affects the sensor response, we measured the response with three different contact speeds *V* = 0.1, 1 and 10 mm/s (10 mm/s is the maximum measurable speed in this setup). The contact point, which corresponds to the center of the indenter, was determined as the position right above the GMR sensor. The sensor responses were investigated with the following procedure: (1) lower the indenter at a speed of *V* until the surface of the sensor descends to a depth of 6 mm, which corresponds to half of the thickness of the dual-layer elastomer; (2) wait for 10 s; (3) raise the indenter to its initial position at a speed of *V*; (4) wait for 10 s; and (5) repeat the above steps 20 times.

**Figure 6:**
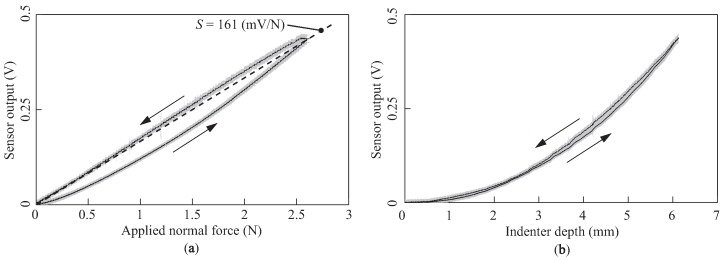
Sensor response curves with a contact speed *V* of 0.1 mm/s: (**a**) the sensor output versus the applied normal force; (**b**) the sensor output versus the indenter depth.

Figure 6 shows the sensor responses versus the applied normal force and indenter depth (i.e., vertical deformation) with a contact speed *V* of 0.1 mm/s, respectively. In both plots, the solid lines and shaded regions indicate the mean value and twice the standard deviation (2σ) of the sensor outputs across 20 trials, respectively. The arrows indicate the directions of the applied load. Each sensor output monotonically increased with the applied normal force and indenter depth. The results in Figure 6b indicate that the sensor response increased quadratically with the indenter depth. Although both curves exhibited hysteresis properties, the effect of the hysteresis was larger for the curve of the sensor response versus the normal force. We calculated the sensitivity *S* (mV/N) during the descending period (dashed line in Figure 6a). The estimated sensitivity was *S* = 161 mV/N or 12.7 mV/kPa (here, we used a cylindrical indenter with a diameter of 10 mm). In addition, the calculated signal-to-noise ratio was 42.2 dB in this setting.

**Figure 7:**
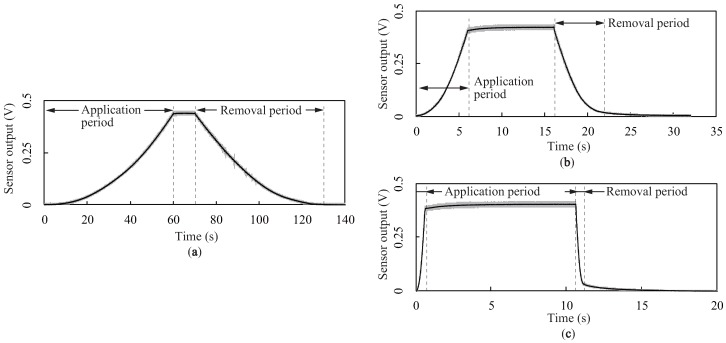
Sensor output versus time for three different contact speeds: (**a**,**b**,**c**) Sensor outputs with three different contact speeds *V* = 0.1, 1, and 10 mm/s, respectively.

In order to evaluate the effect of the contact speed, we measured the sensor outputs with time for three different contact speeds, as shown in Figure 7. The solid lines and shaded regions denote the mean value and twice the standard deviation (2σ) of the sensor outputs across 20 trials, respectively. In each plot, the application and removal periods indicate the periods wherein the load was being applied and removed, respectively. The results demonstrate that the sensor outputs varied similar to a quadratic curve for every speed. On the other hand, for speeds of 1 and 10 mm/s, the outputs slightly increased to their steady outputs after the application period. The differences between the outputs just after the application period and the steady outputs were 15.9 and 29.8 mV for speeds of 1 and 10 mm/s, respectively. Furthermore, the outputs remained at small values even though the loads were completely removed, and these remaining outputs gradually returned to the zero level within a few seconds. The remaining outputs were 18.1 and 29.5 mV for speeds of 1 and 10 mm/s, respectively; thus, a higher speed resulted in a larger remaining output.

### 4.2. Spatial Response

**Figure 8:**
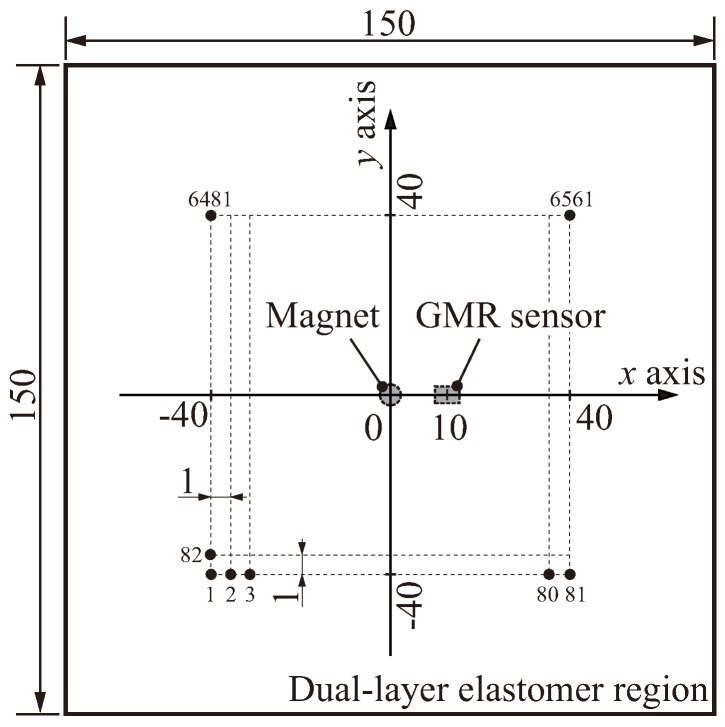
Measurement region and positions of the spatial response. The black dots indicate the center position of the applied load, i.e., the center of the indenter. The indenter was moved from (x,y)=(−40,40) to (40,40) in 1 mm steps based on the index numbers 1, 2, ..., 6,561 of the dots.

To determine an appropriate spatial layout for the proposed sensor for large-area implementation, the spatial response was investigated using the same setup. Figure 8 illustrates the measurement region and positions of the spatial response. The black dots in the figure indicate the center positions where the load was applied, i.e., the center of the indenter. In accordance with the index numbers (1, 2, ..., 6561) of the dots, the indenter moved from *x* = −40 mm to *x* = 40 mm and from *y* = −40 mm to *y* = 40 mm in 1-mm steps along each axis. The positions of the magnet and GMR sensor corresponded to (*x*, *y*) = (0, 0) and (10, 0), respectively.

At each measurement position, the indenter was lowered from the sensor surface $Z_{o}=0$ mm to an arbitrary depth $Z_{max}$, which was set to each of seven different depths $Z_{max}$ = 0, 1, 2, 3, 4, 5 and 6 mm (note that 0 mm means the load was not applied). By subtracting the initial output voltage $V_{o}$ at $Z_{o}$ from the output voltage *V* at $Z_{max}$, we measured the difference in the sensor outputs (i.e., $V-V_{o}$) at all measurement points.

**Figure 9:**
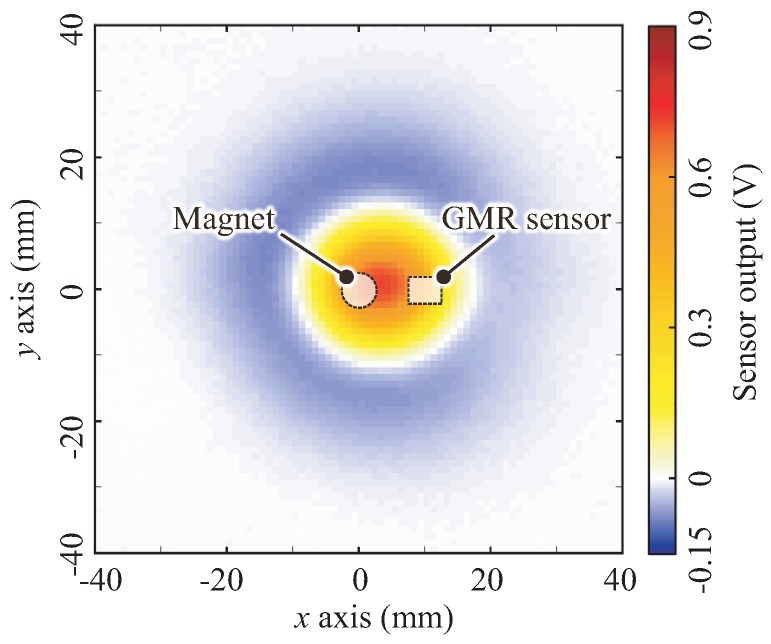
Measured spatial response for a depth of 4 mm. The shape of spatial response exhibits an approximately elliptical Mexican-hat-like bipolar shape whose major axis corresponds to the line connecting the centers of the magnet and GMR sensor.

Figure 9 shows the spatial response measured at a depth of 4 mm ($Z_{max}$ = 4 mm) as one example of the measured data. The horizontal and vertical axes indicate the center position of the indenter along the *x* and *y* axes, respectively. The color depicts the mean value of the sensor outputs across the three trials; warm colors (yellow, orange and red) and blue indicate positive and negative sensor outputs, respectively. In addition, the white region indicates a region with zero output; in other words, the nonsensitive region of the sensor. The shape of the spatial response exhibited an elliptical Mexican-hat-like bipolar shape whose major axis corresponds to the line connecting the centers of the magnet and GMR sensor. The center of the response was approximately ($x_{o},y_{o}$) = (3, 0), i.e., a position between the magnet and the GMR sensor along the line connecting the centers of the magnet and GMR sensor.

**Figure 10:**
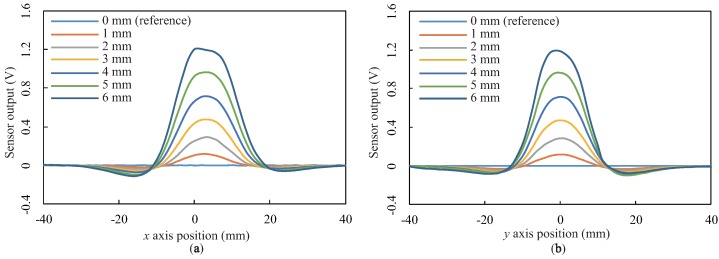
Line profiles of the measured spatial responses with at seven different depths: (**a**,**b**) Sensor outputs along the *x* and *y* axes through the point ($x_{o},y_{o}$) = (3, 0), respectively.

The results for the other depths are summarized in Figure 10 as line profiles along the *x* and *y* axes through the center ($x_{o},y_{o}$) = (3, 0). These responses exhibit Mexican-hat-like bipolar responses for all depths. In addition, the line profiles indicate that the zero-crossing points were almost the same point, i.e., (x,y) = (–10, 0), (18, 0), (3, –12), and (3, 12) in Figure 10, regardless of the depth.

The results demonstrate that the proposed sensor exhibits a Mexican-hat-like bipolar response and not a Gaussian-like response (i.e., a bell-shaped response) generally observed in conventional tactile sensors, e.g., [2,24]. We hypothesized that such a negative response in the bipolar response does not originate from magnetic transduction, but instead from the incompressible characteristics of the BE. Because the BE is made of an incompressible material, bulging of the BE can occur around the edge of the pushed region (see Figure 3, right). This bulging increases the distance between the MRE and the sensing elements and therefore causes the negative response.

**Figure 11:**
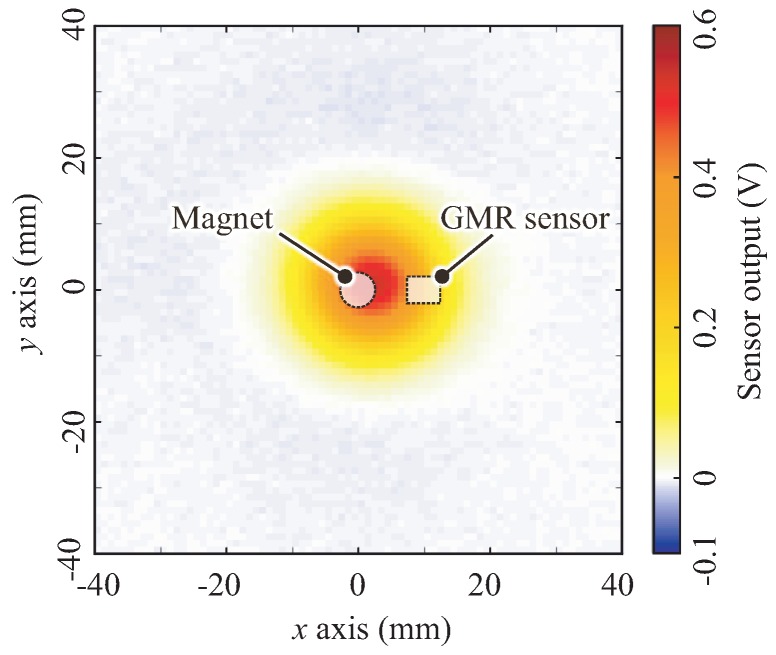
Measured spatial response with a sponge sheet instead of the base elastomer sheet at a depth of 4 mm. The shape of spatial response exhibits an approximately elliptical Gaussian-like shape whose major axis corresponds to the line connecting the centers of the magnet and GMR sensor.

To verify this, we replaced the BE sheet with a compressible soft sponge sheet and measured its spatial response with the same setup and procedure. Figure 11 shows the measured spatial response by using the sponge sheet for a depth of 4 mm ($Z_{max}$ = 4 mm) as one example of the measured data. The result shows that the negative response region clearly disappears and that the spatial response exhibits an elliptical Gaussian-like shape whose major axis corresponds to the line connecting the centers of the magnet and GMR sensor. The center of the response was ($x_{os},y_{os}$) = (2, 0), i.e., a position between the magnet and the GMR sensor along the line connecting the centers of the magnet and GMR sensor.

**Figure 12:**
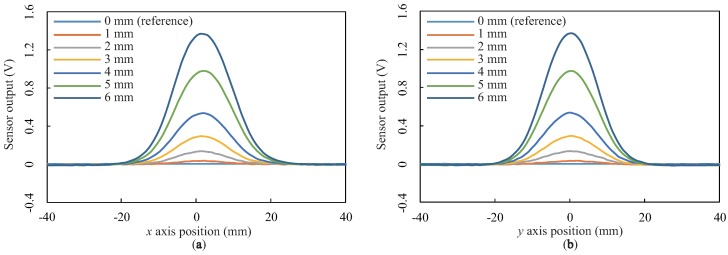
Line profiles of the measured spatial responses with the sponge sheet instead of the base elastomer sheet: (**a**,**b**) Sensor outputs along the *x* and *y* axes through the point ($x_{os},y_{os}$) = (2, 0), respectively.

We again summarize the results as line profiles along the *x* and *y* axes through the center ($x_{os},y_{os}$) = (2, 0). Figure 12 shows the line profiles for the seven different depths with the sponge sheet instead of the BE sheet. As in Figure 11, negative responses were not observed for all depths. Regardless of the depth, the widths of the responses along the *x* and *y* axes were approximately 44 and 40 mm, respectively. These results demonstrate that the negative response is derived from the incompressibility of the BE sheet and not from magnetic transduction.

## 5. Discussion

This section first summarizes the sensor response curves describing the relationship between the sensor outputs and the contact loads and how the contact speed affects the sensor output. Then, we discuss a model of the spatial response shape and whether the bipolar spatial response is useful for tactile information processing.

### 5.1. Sensor Response and Contact Speed

The experiments discussed in Section 4.1 provide the curves showing the fundamental sensor response versus the applied normal force and vertical deformation. The result demonstrated that the applied force and deformation could be estimated from the contact position and shape of the indenter because the curves were either monotonically increasing or decreasing. The curve showing the response versus the indenter depth has a smaller hysteresis compared with the curve showing the response versus the applied force. This is because the sensor response is simply determined by the distance between the MRE and the sensing elements on the PCB. While the applied force is removed, the viscosity of the elastomer generates a residual stress. The repulsive force in response to the indenter becomes weak owing to this residual stress; therefore, the hysteresis becomes larger in the curve showing the sensor response versus the applied force. We also evaluated the sensitivity of the sensor, and found that the estimated sensitivity to the applied force was 161 mV/N with a range of forces of 2.5 N and a signal-to-noise ratio of 42.2 dB in this setup. This value demonstrates that the sensor has a high sensitivity (cf. other sensors listed in [7]), even though the proposed sensor was covered by a highly deformable material.

The results of the experiments utilizing different contact speeds indicate that the sensor outputs similarly varied with the applied load. On the other hand, for contact speeds of 1 and 10 mm/s, the sensor output did not return to the zero level immediately after the load was completely removed. The results for both speeds demonstrate that the sensor output requires a certain time (namely, a relaxation time) to return to its zero level. This remaining output means that the top MRE sheet was still lower than its initial position owing to the viscosity of the elastomer. One area of future work is to compensate for these remaining sensor outputs, e.g., by investigating the deformation dynamics and material properties of the elastomer. Another feasible solution will be to employ a low-viscosity elastic material for the elastomer.

### 5.2. Spatial Response

The experimental results indicate that the proposed sensor has a Mexican-hat-like spatial response, i.e., the sensor has positive and negative response regions. We hypothesized that this negative response was generated by the incompressible characteristics of the dual-layer elastomer. Owing to its incompressibility, the volume of the elastomer is constant before and after applying the load. In addition, the elastomer could not expand to the outside because its four sides were fixed (see Figure 5). Consequently, the BE right below a contact point moves to surrounding region; then, this moved BE pushes the MRE surface up, which decreases the sensor output. The experiments with the soft sponge sheet instead of the BE sheet demonstrate that the negative response region disappeared and that the response shape become an elliptical Gaussian-like shape.

Comparing the two spatial responses (Figure 10 and Figure 12), we also found that the spatial response has a large response region; the diameter of the Mexican-hat-like response along its major axis was approximately 80 mm, and the diameter of the Gaussian-like response along its major axis was approximately 44 mm. A large response region could be generated by employing a thick elastomer that causes surface deformation in a broad region; in particular, this deformation region will become broader by using an incompressible BE, as discussed above.

In conclusion, we can summarize the spatial response of the proposed sensor as follows:

1. The distorted Gaussian-like unipolar spatial response shown in Figure 11 reflects the spatial response generated by the proposed sensing mechanism, i.e., the change in the magnetic field caused by approaching the MRE.
2. The shape of the spatial response is approximately elliptical, whose major axis corresponds to the line connecting the centers of the magnet and GMR sensor.
3. The center of the spatial response ($x_{os}$, $y_{os}$) is at a point that is closer to the magnet along the line connecting the centers of the magnet and GMR sensor.
4. The use of an incompressible elastomer adds a negative response to the unipolar spatial response; then, the entire spatial response becomes a distorted Mexican-hat-like bipolar spatial response, as shown in Figure 9.

The following two subsections describe what functions can fit the measured spatial responses.

#### 5.2.1. Modeling of the Sensor Response Generated by Approaching the MRE

Shimojo [24] found that an elastic covering for a tactile sensor functions as a spatial low-pass filter and that its spatial response exhibits a Gaussian-like shape. Because of the elliptical shapes of the responses in Figure 11 and Figure 12, we employed an elliptical Gaussian function to fit the sensor response generated by approaching the MRE:

(1) $$Res\left(x,y,z\right)=\frac{k(z)}{2\pi \sigma^{2}}\exp\left(-\frac{{\left(x-x_{c}\right)}^{2}+\gamma^{2}{\left(y-y_{c}\right)}^{2}}{2\sigma^{2}}\right)$$

where k(z) is a function of the applied depth *z*, $\left(x_{c},y_{c}\right)$ is the center of the function, $\sigma^{2}$ is the variance and γ is the spatial aspect ratio specifying the ellipticity of the Gaussian function. The results presented in Section 4.1 demonstrate that the sensor output increases quadratically with the applied depth; therefore, we employed a quadratic function $az^{2}+bz+c$ for the function k(z). All parameters were estimated on the basis of a least-squares method, and their estimated values were as follows: $x_{c}$ = 1.774, $y_{c}$ = 0.2067, σ = 7.170, γ = 1.074, *a* = 15.49, *b* = –16.14 and *c* = 4.948. The fitting error $R^{2}$, i.e., the squared error of all measurement points for the seven depths, was 2.2047.

#### 5.2.2. Modeling of the Bipolar Response

The bulging of the incompressible BE sheet added a ring-shaped negative response to the ordinary response described in Equation (1); thus, the entire response becomes a bipolar response (Figure 9). In order to express such a bipolar response, we can practically employ the following two functions: difference of Gaussians (DoG) and Laplacian of Gaussian (LoG) functions. The Mexican-hat-like spatial response is generally fit with these functions.

The DoG function is defined by the difference between two Gaussian functions $G_{1}$ and $G_{2}$ that have different variances and peak values:

(2) $$DoG\left(x,y,z\right)=k\left(z\right)\left(G_{1}\left(x,y\right)-G_{2}\left(x,y\right)\right)$$

(3) $$G_{i}\left(x,y\right)=\frac{1}{2\pi \sigma_{i}^{2}}\exp\left(-\frac{{\left(x-x_{c}\right)}^{2}+\gamma^{2}{\left(y-y_{c}\right)}^{2}}{2\sigma_{i}^{2}}\right)$$

where k(z) is a function of the applied vertical deformation, $\sigma_{i}^{2}$ is the variance, ($x_{c}$, $y_{c}$) is the center position of the DoG function and γ is the spatial aspect ratio that specifies the ellipticity of the DoG function.

On the other hand, the LoG function is defined as a second-order partial differential of a Gaussian function G(x,y):

(4) $$\begin{array}{c} LoG\left(x,y,z\right)=k\left(z\right)\left(\frac{\partial^{2}G\left(x,y\right)}{\partial^{2}x}+\frac{\partial^{2}G\left(x,y\right)}{\partial^{2}y}\right)\\ =-\frac{k(z)}{\pi \sigma^{4}}\left(1-\frac{{\left(x-x_{c}\right)}^{2}+\gamma^{2}{\left(y-x_{c}\right)}^{2}}{2\sigma^{2}}\right)\exp\left(-\frac{{\left(x-x_{c}\right)}^{2}+\gamma^{2}{\left(y-y_{c}\right)}^{2}}{2\sigma^{2}}\right) \end{array}$$

(5) $$G\left(x,y\right)=\frac{1}{2\pi \sigma^{2}}\exp\left(-\frac{{\left(x-x_{c}\right)}^{2}+\gamma^{2}{\left(y-y_{c}\right)}^{2}}{2\sigma^{2}}\right)$$

where k(z) is a function of the applied vertical deformation, $\sigma^{2}$ is the variance, ($x_{c}$, $y_{c}$) determines the center position of the LoG function and γ is the spatial aspect ratio that specifies the ellipticity of the LoG function.

**Figure 13:**
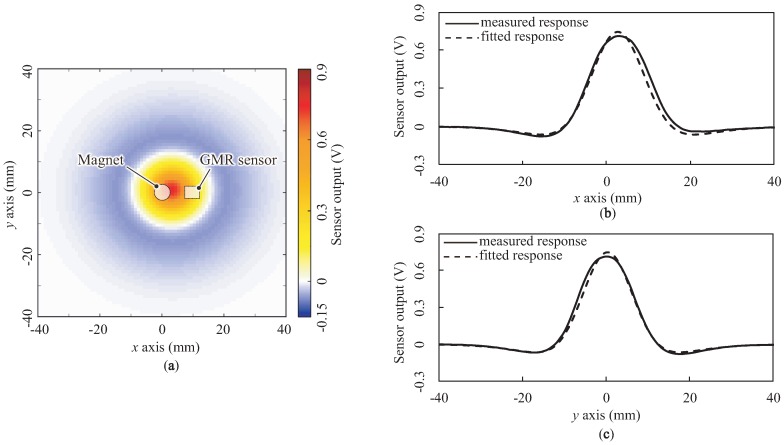
Comparison between the measured and fitted responses for a depth of 4 mm: (**a**) two-dimensional fitted response and (**b**,**c**) line profiles along the *x* and *y* axes through the center ($x_{o},y_{o}$) = (0, 0), respectively.

We conducted a $\chi^{2}$ fitting in order to compare the fitting accuracies of these two functions. The calculated values of the DoG function were as follows: $x_{c}$ = 2.69, $y_{c}$ = 0.369, $\sigma_{1}$ = 6.32, $\sigma_{2}$ = 14.6, γ = 1.06, *a* = 4.07, *b* = 41.5 and *c* = 0.0911; the $\chi^{2}$ value was $3.76\times 10^{6}$. In contrast, the calculated values of the LoG function were as follows: $x_{0}$ = 2.61, $y_{o}$ = 0.468, σ = 8.82, γ = 1.02, *a* = –282, *b* = $-2.00\times 10^{1}$ and *c* = –12.5; the $\chi^{2}$ value was $6.18\times 10^{6}$. $\chi^{2}$ was smaller for the DoG function; thus, we concluded that the DoG function is a practically sufficient fitting of the spatial response of the proposed sensor. Figure 13 shows a comparison between the measured and fitted responses for a depth of 4 mm as one example of the fitted response. Figure 13a depicts the two-dimensional fitted response (cf. Figure 9). Both plots (Figure 13b,c) show the line profiles along the *x* and *y* axes through the center ($x_{o},y_{o}$) = (0, 3). The solid and dotted lines indicate the measured and fitted responses, respectively. These plots also demonstrate that the DoG function can express the spatial response of the proposed sensor.

### 5.3. Usefulness of the Mexican-Hat-Like Spatial Response for Tactile Information Processing

The experimental results show that the proposed sensor has a Mexican-hat-like bipolar spatial response. Note that the BE sheet in the proposed sensor can be easily replaced with any material possessing a low magnetic permeability, as done with the soft sponge sheet employed in this study. If the negative response is unnecessary for a specific application, the BE sheet can be replaced with a compressible soft material. Accordingly, we can choose whether the negative response is utilized in accordance with real tasks or applications. In this section, we discuss whether the negative response is useful for tactile information processing or not.

Such a spatial response can be fit with a DoG function, which is commonly known as an edge-enhancement filter in computer vision. This means that the sensor could function as a spatial filter and extract DoG-like features at the hardware level. Therefore, the proposed sensor may be able to provide edge-enhanced tactile images (or edge-enhancement features) by implementing many pairs of magnets and GMR sensors arranged in an array. In addition, it is widely known that humans utilize edge information when processing tactile information [32]. We believe that such tactile edge enhancement features will be useful for tactile information processing in the field of robotics.

One example employing a DoG function for tactile processing is a scale-invariant feature transform (SIFT) algorithm, which is used to extract features in computer-vision applications. The SIFT requires various scaling DoG features and is employed in object-shape recognition tasks using a tactile sensory signal [33]. Typically, spatial filtering is accompanied with high computational costs; however, the proposed sensor has the potential to perform filtering processes with DoG features in hardware if the proposed sensor is able to vary its spatial response.

The parameters of the BE, such as the thickness and stiffness, are thought to determine the shape of the spatial response. An area of future research is to investigate how these parameters determine the shape and to develop a design procedure for creating an arbitrary spatial response for the sensor.

## 6. Conclusions

We proposed a flexible tactile sensor using a dual-layer elastomer and magnet and investigated the sensor responses with different contact speeds and the spatial responses. The first experiments demonstrated that the sensor has a high sensitivity (approximately 161 mV/N with a signal-to-noise ratio of 42.2 dB) and that the sensor can respond to the applied load, even with a high contact speed; however, a high contact speed induces a relaxation time in the sensor output. The second experiment found that the sensor has an elliptical Mexican-hat-like bipolar spatial response whose major axis corresponds to the line connecting the centers of the magnet and magnetic transducer and that employing a incompressible dual-layer elastomer results in a negative response in the bipolar response. We then demonstrated that this bipolar spatial response can be practically fit with an elliptical DoG function. This spatial-filter-like response indicates that the proposed sensor may be able to function as a spatial filter at the hardware level.

In this paper, we evaluated the output with a single-magnet and GMR-sensor pair. Hence, the next step is to focus on the implementation of multiple magnets and GMR sensors. We believe that the combination of outputs from these pairs of devices will enable the estimation of the contact position, the applied vertical deformation and the shape of the contacted object. We will also mount the proposed sensor on the body of a humanoid robot for further testing.

References

## References

[1] Minato T., Yoshikawa Y., Noda T., Ikemoto S., Ishiguro H., Asada M. CB^2^: A Child Robot with Biomimetic Body for Cognitive Developmental Robotics. Proceedings of the 2007 7th IEEE-RAS International Conference on Humanoid Robots (Humanoids).

[2] Maiolino P., Maggiali M., Cannata G., Metta G., Natale L. (2010). A Flexible and Robust Large Scale Capacitive Tactile System for Robots. IEEE Sens. J..

[3] Argall B.D., Billard A.G. (2010). Survey of Tactile Human—Robot Interactions. Robot. Auton. Syst..

[4] Kawasetsu T., Horii T., Ishihara H., Asada M. Difference of Gaussian Like Feature Enhances Object Classification Accuracy in Magnetorheological Elastomer-Gel Tactile Sensor. Proceedings of the 2016 IEEE International Conference Humanoid Robots (Humanoids) Workshop on Tactile Sensing for Manipulation: New Progress and Challenges.

[5] Kawasetsu T., Horii T., Ishihara H., Asada M. (2016). Deformation Response of a Magnetic Type Tactile Sensor with a Two-Layered Surface Made of a Non-magnetic and a Magnetorheological Elastomer. J. Jpn. Soc. Appl. Electromagn. Mech..

[6] Kawasetsu T., Horii T., Ishihara H., Asada M. Magnetorheological Elastomer-Gel Tactile Sensor with an Electromagnet. Proceedings of the 2017 International Conference on Robotics and Automation (ICRA) Workshop on the Robotic Sense of Touch: From Sensing to Understanding.

[7] Dahiya R.S., Metta G., Valle M., Sandini G. (2010). Tactile Sensing—From Humans to Humanoids. IEEE Trans. Robot..

[8] Dahiya R.S., Mittendorfer P., Valle M., Cheng G., Lumelsky V.J. (2013). Directions Toward Effective Utilization of Tactile Skin: A Review. IEEE Sens. J..

[9] Hosoda K., Tada Y., Asada M. (2006). Anthropomorphic Robotic Soft Fingertip with Randomly Distributed Receptors. Robot. Auton. Syst..

[10] Song A., Han Y., Hu H., Li J. (2014). A Novel Texture Sensor for Fabric Texture Measurement and Classification. IEEE Trans. Instrum. Meas..

[11] Hu H., Han Y., Song A., Chen S., Wang C., Wang Z. (2014). A Finger-Shaped Tactile Sensor for Fabric Surfaces Evaluation by 2-Dimensional Active Sliding Touch. Sensors.

[12] Futai N., Matsumoto K., Shimoyama I. (2004). A Flexible Micromachined Planar Spiral Inductor for Use as an Artificial Tactile Mechanoreceptor. Sens. Actuators A.

[13] Goka M., Nakamoto H., Takenawa S. A Magnetic Type Tactile Sensor by GMR Elements and Inductors. Proceedings of the 2010 IEEE/RSJ International Conference on Intelligent Robots and Systems (IROS).

[14] Kim J., Lee M., Shim H.J., Ghaffari R., Cho H.R., Son D., Jung Y.H., Soh M., Choi C., Jung S. (2014). Stretchable Silicon Nanoribbon Electronics for Skin Prosthesis. Nat. Commun..

[15] Lee S., Reuveny A., Reeder J., Lee S., Jin H., Liu Q., Yokota T., Sekitani T., Isoyama T., Abe Y. (2016). A Transparent Bending-Insensitive Pressure Sensor. Nat. Nanotechnol..

[16] Wang X., Dong L., Zhang H., Yu R., Pan C., Wang Z.L. (2015). Recent Progress in Electronic Skin. Adv. Sci..

[17] Drimus A., Kootstra G., Bilberg A., Kragic D. (2014). Design of a flexible tactile sensor for classification of rigid and deformable objects. Robot. Auton. Syst..

[18] Lepora N.F., Martinez-Hernandez U., Prescott T.J. Active touch for robust perception under position uncertainty. Proceedings of the 2013 IEEE International Conference on Robotics and Automation (ICRA).

[19] Buescher G.H., Koiva R., Schuermann C., Haschke R., Ritter H.J. (2015). Flexible and Stretchable Fabric-Based Tactile Sensor. Robot. Auton. Syst..

[20] Shimojo M., Araki T., Teshigawara S., Ming A., Ishikawa M. A Net-Structure Tactile Sensor Covering Free-form Surface and Ensuring High-Speed Response. Proceedings of the 2007 IEEE/RSJ International Conference on Intelligent Robots and Systems (IROS).

[21] Mukai T., Onishi M., Odashima T., Hirano S., Luo Z. (2008). Development of the Tactile Sensor System of a Human-Interactive Robot “RI-MAN”. IEEE Trans. Robot..

[22] Ohmura Y., Kuniyoshi Y., Nagakubo A. Conformable and Scalable Tactile Sensor Skin for Curved Surfaces. Proceedings of the 2006 IEEE International Conference on Robotics and Automation (ICRA).

[23] Mittendorfer P., Cheng G. (2011). Humanoid Multimodal Tactile Sensing Modules. IEEE Trans. Robot..

[24] Shimojo M. Spatial Filtering Characteristic of Elastic Cover for Tactile Sensor. Proceedings of the 1994 IEEE International Conference on Robotics and Automation (ICRA).

[25] Jamone L., Natale L., Metta G., Sandini G. (2015). Highly Sensitive Soft Tactile Sensors for an Anthropomorphic Robotic Hand. IEEE Sens. J..

[26] Youssefian S., Rahbar N., Torres-Jara R. (2014). Contact Behavior of Soft Spherical Tactile Sensors. IEEE Sens. J..

[27] Tomo T.P., Somlor S., Schmitz A., Jamone L., Huang W., Kristanto H., Sugano S. (2016). Design and Characterization of a Three-Axis Hall Effect-Based Soft Skin Sensor. Sensors.

[28] Liu Y., Han H., Liu T., Yi J., Li Q., Inoue Y. (2016). A Novel Tactile Sensor with Electromagnetic Induction and Its Application on Stick-Slip Interaction Detection. Sensors.

[29] Kamiyama K., Vlack K., Mizota T., Kajimoto H., Kawakami K., Tachi S. (2005). Vision-Based Sensor for Real-Time Measuring of Surface Traction Fields. IEEE Comput. Graph. Appl..

[30] Takagi S., Yamamoto Y., Ohka M., Yussof H., Abdullah S.C. (2015). Sensitivity-Enhancing All-in-Type Optical Three-Axis Tactile Sensor Mounted on Articulated Robotic Fingers. Procedia Comput. Sci..

[31] Yamaguchi A., Atkeson C.G. Combining Finger Vision and Optical Tactile Sensing: Reducing and Handling Errors While Cutting Vegetables. Proceedings of the 2016 IEEE-RAS International Conference on Humanoid Robots (Humanoids).

[32] Pruszynski J.A., Johansson R.S. (2014). Edge-Orientation Processing in First-Order Tactile Neurons. Nat. Neurosci..

[33] Luo S., Mou W., Althoefer K., Liu H. (2015). Novel Tactile-SIFT Descriptor for Object Shape Recognition. IEEE Sens. J..

